# Retraction: The Role for HNF-1β-Targeted Collectrin in Maintenance of Primary Cilia and Cell Polarity in Collecting Duct Cells

**DOI:** 10.1371/journal.pone.0286174

**Published:** 2023-05-18

**Authors:** The PLOS ONE Editors

Following the publication of this article [1], concerns were raised regarding results presented in Figures 1, 2, 5, and 6. Specifically,

- When adjusting the color levels to visualize the background, there appear to be irregularities in the background around the bands presented in the Figure 1A EMSA blots.
- When adjusting the color levels to visualize the background, there appear to be irregularities in the background of the Figure 5A results, suggesting that these results may have been obtained from spliced blots.
- When adjusting the color levels to visualize the background, there appear to be irregularities in the background around the bands presented in the Figure 5B results.
- The Figure 6A Con-siRNA panel appears similar to the Figure 6A Collectrin stable panel when rotated 180°. In addition, these two panels partially overlap with the Figure 6A mIMCD3 results.
- When rotated 180°, the Figure 6D mIMCD3 results appear more similar than expected to the Figure 6D Collectrin stable results.

The corresponding author provided underlying data for the Figure 1A EMSA blots. Although the bands on these blots did not appear to be a perfect match with the published figure, they did appear to support the published results. The underlying data for Figures 5A and 5B are no longer available.

Regarding Figure 6, the corresponding author explained that the wrong panels were used during the preparation of Figures 6A and 6D, and provided replacement data. However, the corresponding author’s explanation does not clarify the concerns pertaining to the Figure 6D panels.

In addition to the concerns listed above, during the assessment of the underlying data provided for the Figure 2A blots, it appeared that the published Snapin results in lanes 2, 3, and 4 matched the underlying data in lanes 4, 3, and 2 respectively when the underlying data was flipped horizontally, suggesting that the Figure 2A Snapin results may have been mislabelled. Furthermore, the corresponding author indicated that the incorrect Collectrin panel was used during the preparation of this figure and provided replacement data.

The underlying data for Figures 1, 2, 5C, and 6 were provided for editorial review. Furthermore, the corresponding author stated that the underlying data for Figures 3, 4, 7, and 8 remain available upon request, but that the underlying data for Figures 5A and 5B are no longer available.

In light of the concerns affecting multiple figure panels that question the integrity of these data, the *PLOS ONE* Editors retract this article.

JW, AY, II, JE, KY, SA, YSK, and HM agreed with the retraction, and apologized for the issues with the published article. YZ, KF, QY, TH, PI, HZ, and HW either did not respond directly or could not be reached.
